# On the road to explainable AI in drug-drug interactions prediction: A systematic review

**DOI:** 10.1016/j.csbj.2022.04.021

**Published:** 2022-04-19

**Authors:** Thanh Hoa Vo, Ngan Thi Kim Nguyen, Quang Hien Kha, Nguyen Quoc Khanh Le

**Affiliations:** a Master Program in Clinical Genomics and Proteomics, College of Pharmacy, Taipei Medical University, Taipei 110, Taiwan; b School of Nutrition and Health Sciences, College of Nutrition, Taipei Medical University, Taipei 11031, Taiwan; c International Master/Ph.D. Program in Medicine, College of Medicine, Taipei Medical University, Taipei 110, Taiwan; d Professional Master Program in Artificial Intelligence in Medicine, College of Medicine, Taipei Medical University, Taipei 106, Taiwan; e Research Center for Artificial Intelligence in Medicine, Taipei Medical University, Taipei 106, Taiwan; f Translational Imaging Research Center, Taipei Medical University Hospital, Taipei 110, Taiwan

**Keywords:** Explainable artificial intelligence, Drug-drug interaction, Machine learning, Deep learning, Chemical structures, Natural language processing

## Abstract

Over the past decade, polypharmacy instances have been common in multi-diseases treatment. However, unwanted drug-drug interactions (DDIs) that might cause unexpected adverse drug events (ADEs) in multiple regimens therapy remain a significant issue. Since artificial intelligence (AI) is ubiquitous today, many AI prediction models have been developed to predict DDIs to support clinicians in pharmacotherapy-related decisions. However, even though DDI prediction models have great potential for assisting physicians in polypharmacy decisions, there are still concerns regarding the reliability of AI models due to their black-box nature. Building AI models with explainable mechanisms can augment their transparency to address the above issue. Explainable AI (XAI) promotes safety and clarity by showing how decisions are made in AI models, especially in critical tasks like DDI predictions. In this review, a comprehensive overview of AI-based DDI prediction, including the publicly available source for AI-DDIs studies, the methods used in data manipulation and feature preprocessing, the XAI mechanisms to promote trust of AI, especially for critical tasks as DDIs prediction, the modeling methods, is provided. Limitations and the future directions of XAI in DDIs are also discussed.

## Highlights

• A systematic review on applications of explainable AI in drug-drug interaction prediction.
• Review is conducted on a comprehensive set of 94 papers from five prestigious databases.
• Discussions on the promises and challenges of explainable AI algorithms for drug-drug interaction prediction.

## Graphical abstract

**Figure:**
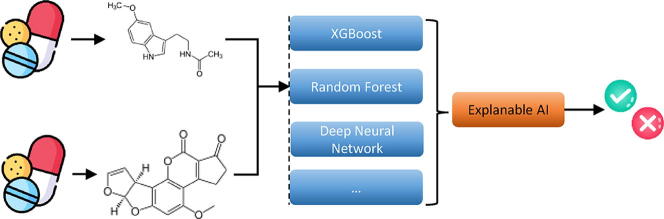

## 1 Introduction

Drug-drug interactions (DDIs) usually happen in polypharmacy instances when the effects of a drug alter that of others in a combined regimen. In treatment, preferably, synergistic action and therapeutic benefit are expected. However, in multi-diseases treatment, adverse drug events (ADEs) that cause toxicity or reduced treatment effect may also inevitably happen. These can eventually lead to increased morbidity and mortality in patients [1], [2], [3]. In addition, an increased number of recently frequent launches and approval of new drugs and indications in marketed medicines introduces more possible DDIs occurrences [4], [5]. However, wet-lab experiments for verifying DDIs can drain researchers' time and resources and make it difficult for numerous and regular adoptions. Therefore, artificial intelligence (AI) models have been applied to predict DDIs [6], [7], [8], [9]. These models have been continuously studied and improved along with the expansion and completeness of drug-database resources to support clinical decisions.

However, since the introduction of AI-models in DDIs recognition, many efforts have been applied to boost the predictive power of algorithms by putting forward more complex systems, turning these models into those called “black-box AI” that hinder the ability of users to explain how these models work [10]. Specifically, higher performance models are associated with more sophisticated systems, but lower performance tools with simple approaches are easier to comprehend [11]. Despite various benefits given by widespread industrial adoption of machine learning (ML) models, a critical domain as healthcare should be taken more seriously due to its immense value to humans. Additionally, from a human-oriented research angle, the ambiguity of complicated models in making predictive decisions hamper its successful adoption in medical settings as unable-to-interpreted systems are difficult to be trusted. Since the fundamental application of AI in drug treatment must first do with DDIs, explainable DDIs-AI models are pivotal for clinicians and patients to understand and trust their prediction. In response, the ignition of the field explainable artificial intelligence (XAI), which concentrates on methods to interpret ML models, has revived over recent years. XAI can facilitate clinical applications of DDIs prediction models regarding their requirement of robust yet human-understandable systems to provide clear justifications and promote safety, reliability, and transparency.

This review focuses on the advances of recently developed DDIs prediction models regarding their data manipulation technique, feature selection process, modeling approach, XAI method, and the challenge of assuring explainability and transparency of DDIs-prediction models without compromising the predictive power of these systems.

## 2 Study selection

The Preferred Reporting Items for Systematic Reviews and Meta-Analyses (PRISMA) guideline was referenced when conducting literature reviewing [12]. We searched five electronic databases up to December 2021: Cochrane Library, PubMed, EMBASE, IEEE, and Scopus. The search strategy combined the Medical Subject Headings terms and free terms “drug drug interaction” or “drug-drug interaction”, in combination with “artificial intelligence” or “machine learning” or “deep learning” or “neural network” and “prediction model”.

The eligibility criteria consisted of DDI predictive models that were built up using ML - and/or DL-based algorithms. The articles were screened and selected independently by two reviewers (N.T.K.N and H.T.V.), and disagreements were resolved by the third reviewer (N.Q.K.L.). All the retrieved publications were entered into reference-manager software (EndNote X9, Excel 2018).

**Fig. 1:**
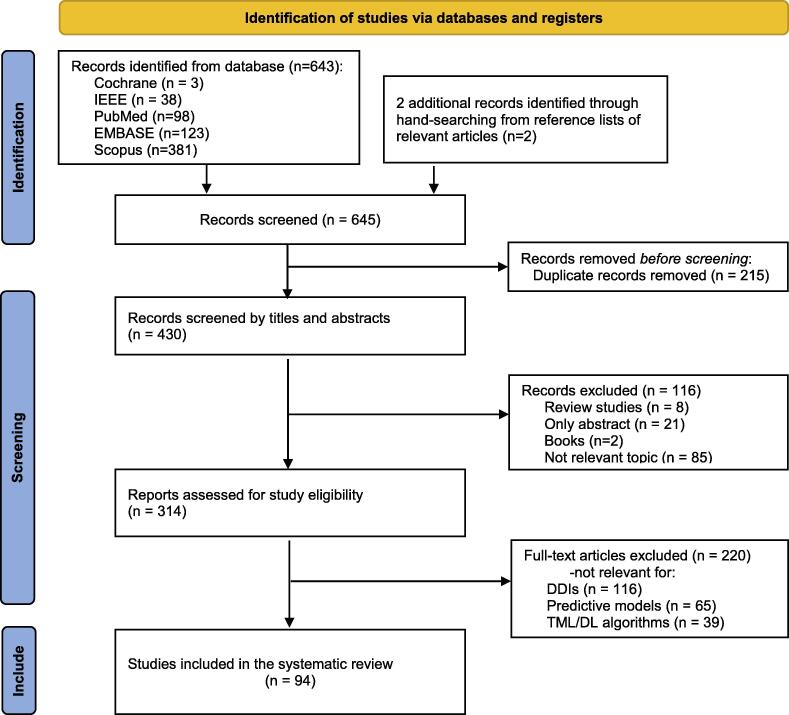
PRISMA diagram showing our literature strategy search.

We identified 643 records through Cochrane Library, IEEE, PubMed, EMBASE, Scopus database,  and two records from reference lists of review paper. After removing 215 duplicates, 116 records were excluded according to the screening of titles and abstracts. Of 314 remaining research studies, 220 studies were removed after evaluating the selection criteria: (1) related to DDIs, (2) related to predictive model, (3) focused on ML or/and DL. As a result, we had 94 different research studies. Fig. 1 shows the flow diagram of the systematic search. Table 1 shows the detailed information of 94 selected studies.

**Table 1:** Input data type of all papers reviewed in this study.

No.	Method	Authors	Year	Input data	Algorithm	Performance
1	TML	Cheng et al. [6]	2014	structure	SVM	AUC ∼ 0.565 to 0.666
2		Hunta et al. [54]	2017	structure	SVM	AUC = 0.901
3		Deepika et al. [81]	2018	structure	meta classifier	F1-score = 0.909
4		Dhami et al. [51]	2018	structure	kernel learning	Accuracy > 0.7
5		Mahadevan et al. [48]	2019	structure	ensemble learning	Accuracy > 0.9
6		Zhang et al. [70]	2019	structure	ensemble learning	AUC = 0.9951
7		Song et al. [84]	2019	structure	SVM	AUC > 0.97
8		Qian et al. [60]	2019	structure	gradient boosting	AUC = 0.689
9		Wang et al. [85]	2020	structure	SVM	AUC = 0.985
10		Rohani et al. [79]	2020	structure	integrated similarity-constrained matrix factorization	F1-score = 0.885
11		Zhan et al. [92]	2020	structure	Bayesian networks coupled with level-wise algorithm	Precision = 0.5445
12		Huang et al. [141]	2020	structure	Chemical Sequential Pattern Mining	AUC = 0.91
13		Hung et al. [94]	2021	structure	ensemble learning	Accuracy = 0.7
14		Dang et al. [49]	2021	structure	XGBoost	F1-score = 0.65
15		Patrick et al. [72]	2021	structure	ensemble learning	AUC > 0.9
16		Dewulf et al. [142]	2021	structure	combined multi-regression	AUC = 0.843
17		Mei et al. [83]	2021	structure	L2-regularized logistic regression	AUC = 0.9884
18		Thomas et al. [17]	2011	text	ensemble learning	F1-score = 0.657
19		Minard et al. [143]	2011	text	SVM	F1-score = 0.5965
20		Garcia-Blasco et al. [16]	2011	text	RF	F1-score = 0.6341
21		Boyce et al. [87]	2012	text	SVM	F1-score = 0.859
22		Zhang et al. [89]	2012	text	single kernel	AUC = 0.924
23		Hailu et al. [19]	2013	text	SVM	F1-score = 0.5
24		Bjorne et al. [18]	2013	text	Turku Event Extraction System	F1-score = 0.59
25		Bobic et al. [95]	2013	text	LibLINEAR, perceptron Naïve Bayes	F1-score = 0.704
26		Yan et al. [73]	2013	text	Drug-Entity-Topic	AUC = 0.96
27		Zhang et al.[90]	2015	text	Label Propagation	AUC = 0.864
28		Ben Abacha A et al.[38]	2015	text	Hybrid CRF based	F1-score = 0.6398
29		Bokharaeian et al. [31]	2016	text	bag of word kernel	sign test p-value < 0.0001
30		Mahendran et al. [144]	2016	text	bag of word	F1-score = 0.769
31		Zhang et al. [28]	2017	text	ensemble learning	–
32		Celebi et al. [75]	2019	text	RF	AUC = 0.91
33		Javed et al. [82]	2021	text	RF	Accuracy = 0.954
34		Xie et al. [42]	2021	text	LR	Precision = 0.9
35	DL	Polak et al. [59]	2005	structure	ANN	AUC = 0.82
36		Herrero-Zazo et al. [53]	2016	structure	ANN	F1-score = 0.64
37		Ryu et al. [7]	2018	structure	DNN	Accuracy = 0.924
38		Lee et al. [55]	2018	structure	RWR coupled with KNN	AUC = 0.67
39		Karim et al. [145]	2019	structure	Graph Auto-Encoders	AUC = 0.98
40		Rohani et al. [77]	2019	structure	ANN	AUC from 0.954 to 0.994
41		Lee et al. [80]	2019	structure	auto-encoder coupled with a deep feed-forward network	Accuracy > 0.95
42		Hou et al. [45]	2019	structure	DNN	AUC = 0.942
43		Liu et al. [146]	2019	structure	multilayer bidirectional LSTM	F1-score = 0.7243
44		Karim et al. [66]	2019	structure	Convolutional-LSTM network	F1-score = 0.92
45		Shukla et al. [97]	2019	structure	convolutional mixture density RNN	Accuracy = 0.982
46		Deng et al. [50]	2020	structure	Multi DNN	F1-score = 0.7585
47		Lin et al. [68]	2020	structure	Knowledge Graph Neural Network	AUC = 0.9912
48		Zhang et al. [62]	2020	structure	multi-modal deep auto-encoders	F1-score = 0.8498
49		Feng et al. [52]	2020	structure	GCN-DNN	F1-score = 0.84
50		Shankar et al. [71]	2020	structure	ANN	AUC = 0.69
51		Masumshah et al. [102]	2021	structure	ANN	F1-score = 0.936
52		Zitnik et al. [74]	2021	structure	spectral convolution	AUC = 0.928
53		Lin et al. [56]	2021	structure	CNNs, auto-encoders with Siamese network	F1-score = 0.9117
54		Schwarz et al. [61]	2021	structure	multi-modal neural network	AUPRC from 0.77 to 0.92
55		Luo et al. [57]	2021	structure	graph convolutional auto-encoder network	–
56		Nyamabo et al. [65]	2021	structure	graph neural network	AUC = 0.9838
57		Chen et al. [107]	2021	structure	integrated modules neural network	AUC = 0.9994
58		Pathak et al. [29]	2013	text	Linked Data	–
59		Zhao et al. [34]	2016	text	Syntax CNN	F1-score = 0.686
60		Liu et al. [41]	2016	text	CNN	F1-score = 0.6975
61		Quan et al. [109]	2016	text	multichannel CNN	F1-score = 0.702
62		Zhang et al. [24]	2016	text	SVM	F1-score = 0.8497
63		Suárez-Paniagua et al. [105]	2017	text	CNN	F1-score = 0.6198
64		Zheng et al. [130]	2017	text	RNN with LSTM units	F1-score = 0.773
65		Kavuluru et al. [123]	2017	text	character-level RNNs	F1-score = 0.7081
66		Wang et al. [147]	2017	text	RNN with LSTM and an attention mechanism	F1-score = 0.715
67		Yi et al. [129]	2017	text	RNN	F1-score = 0.722
68		Jiang et al. [127]	2017	text	skeleton-LSTM	F1-score = 0.714
69		Li et al. [96]	2017	text	relation classification framework based on topic modeling	F1-score = 0.48
70		Wang et al. [120]	2017	text	LSTM	F1-score = 0.72
71		Zhang et al. [33]	2017	text	hierarchical RNN	F1-score = 0.729
72		Xu et al. [26]	2018	text	bidirectional LSTM network	F1-score = 0.7115
73		Sun et al. [112]	2018	text	Deep CNN	F1-score = 0.845
74		Lim et al. [21]	2018	text	recursive neural network	F1-score = 0.838
75		Zhou et al. [126]	2018	text	BiLSTM	F1-score = 0.7299
76		Zhang et al. [20]	2018	text	RNN-CNN	F1-score = 0.648
77		Zitnik et al. [113]	2018	text	spectral convolution	AUC = 0.928
78		Paniagua et al. [104]	2018	text	CNN	F1-score = 0.6456
79		Hou et al. [100]	2018	text	LSTM- DNN	F1-score = 0.875
80		Sahu et al. [119]	2018	text	LSTM	F1-score = 0.6939
81		Zhang et al. [93]	2019	text	variational autoencoder	F1-score = 0.579
82		Xiong et al. [114]	2019	text	combined GCNN and BiLSTM	F1-score = 0.77
83		Liu et al. [146]	2019	text	non-linear unsupervised neural network + RF	F1-score = 0.8498
84		Sun et al. [43]	2019	text	recurrent hybrid CNN	F1-score = 0.7548
85		Shtar et al. [101]	2019	text	ensemble-based classifier	AUC 0.807 to 0.990
86		Xu et al. [25]	2019	text	full-attention network	F1-score = 0.712
87		Wu et al. [108]	2020	text	stacked bidirectional GRU + CNN	F1-score = 0.75
88		Zhu et al. [36]	2020	text	bidirectional transformer + BiGRU	F1-score = 0.809
89		Liu et al. [27]	2020	text	stacked autoencoders + weighted SVM	–
90		Park et al. [32]	2020	text	Attention-based Graph Convolutional Networks	F1-score = 0.7686
91		Zaikis et al. [128]	2020	text	stacked Bi-LSTM + CNN	–
92		Allahgholi et al. [23]	2020	text	ANN	Accuracy = 0.954
93		Warikoo et al. [35]	2020	text	Lexically-aware Transformer-based BERT	F1-score = 0.645
94		Fatehifar et al. [40]	2021	text	LSTM	F1-score = 0.783

TML: traditional machine learning, DL: deep learning, '-'the information was not reported in the original paper.

**Fig. 2:**
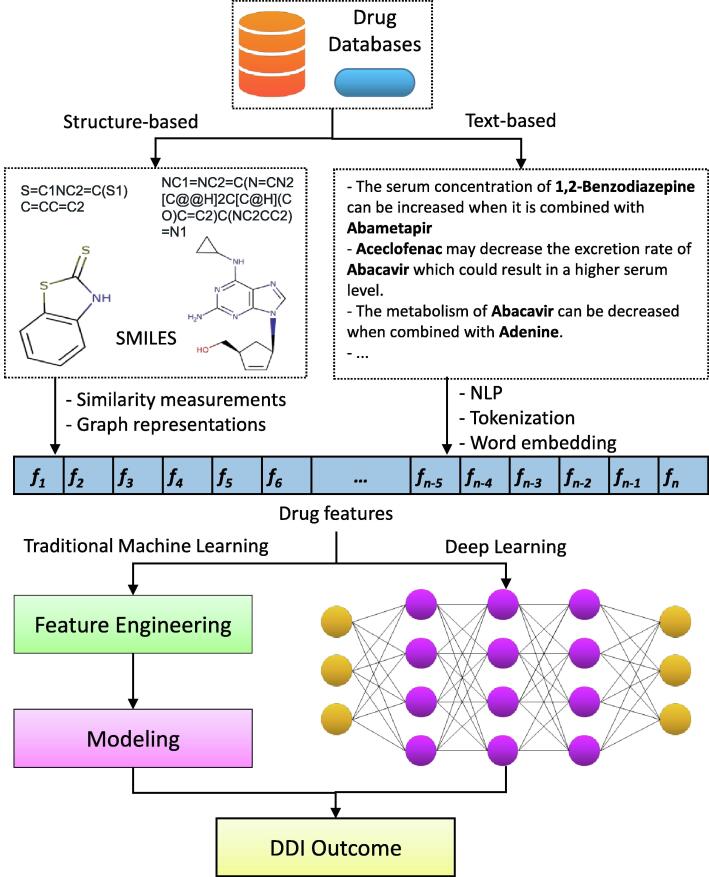
Overall workflow of traditional ML and DL for DDIs prediction.

**Fig. 3:**
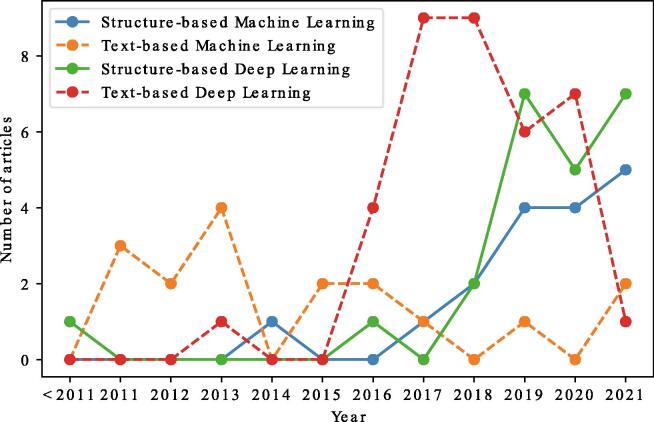
Evolution of DDI prediction models separated by different input data and algorithms.

The flowchart of AI-based DDI prediction model is illustrated in Fig. 2. From the whole flowchart, we would like to conduct our review based on two main aspects: input data (DDIs extraction and feature preprocessing) and AI algorithms (traditional machine learning and deep learning). The evolution of DDI prediction models separated by these two aspects is also shown in Fig. 3.

## 3 Dataset, input data, and features for AI-DDIs studies

In response to the growing number of pharmaceutical drugs entering the market over the past decades, many drug-related information databases have been updating and expanding to facilitate DDIs prediction [13], [14], [15]. Generally, most DDIs studies referred to datasets from DDIExtraction 2011 [16], [17], DDIExtraction 2013 [18] and DrugBank database [19]. These public sources provide various types of drugs' characteristics and DDIs events to leverage AI approaches for DDIs discovery. The quantitative information about the DDIs is a necessary part of creating the described system. The data record format usually has binary characters encoded as 1 if there is an interaction between two drugs and 0 if there is a lack of known interaction.

Depending on the DDIs features-based view of different approaches, appropriate data extraction and feature preprocessing methods for DDIs prediction tasks can be applied.

### 3.1 DDIs information retrieved from text-based sources

This method involves extracting DDIs information in the form of biomedical text, especially in scientific literature since these sources represent valuable information for the retrieval of knowledge about the interaction between drugs. The amount of biomedical literature, which holds a vast amount of DDIs, has been growing over the past years and facilitating many DDIs extracting studies [20], [21], [22]. Aside from studies using public available DDI corpus [23], [24], some studies have also used additional user-generated content to compensate for the limits of delayed updates of the medical database [25], [26]. In addition, multi-information sources DDI corpora have been constructed based on useful information from FDA adverse event reports [27], [28], electronic health records (EHRs) [29], [30], or by following specific annotation guidelines [31] to construct corpus for DDIs extracting.

In these DDIs extraction approaches, feature preprocessing is essential. In detail, tokenization and lower casing are the first vital steps in reducing the sparsity of feature space. Also, many dimensionally reduction text preprocessing techniques have been used for DDIs extraction. Some compression techniques such as sentence pruning [32] and anaphora resolution have been applied [33]; Zhao used syntax word embedding strategy [34] instead of the common word embedding technique, some used Bidirectional Encoder Representations from Transformers (BERT) that relies on attention mechanism to capture high-quality contextual information [35], [36]. The domain-specific ontologies approach attempted to use ancestors' sequences in the ontology to represent each entity [37]. Bokharaeian *et al.* [31] proposed clause dependency features to improve the relation extraction performance. Also, Ben Abacha et al. [38] used the CRF-based algorithm trained by a set of linguistic and semantic features for the drug name recognition. Later, the DDIs extraction task was built on a hybrid method of both feature-based and kernel-based machine learning approaches. Moreover, the imbalanced class distribution problem has also been considered in many articles since this issue can diminish the power of classification [39], [40]. Liu et al. used several rules to filter negative instances [41]; others added random negative sampling as part of the active learning algorithm to deal with the imbalanced issue [42] or use focal loss function to mitigate against this problem [43].

### 3.2 Molecule-based input data and feature preprocessing for DDIs prediction

Usually, DDIs studies utilize chemical, molecular, and pharmacological properties information to elucidate drug interactions insights. In detail, the chemical properties of drugs are typically described via the simplified molecular-input line-entry system (SMILES). This flexible chemical notation allows the generation of computer-feedable input [44]. These SMILES structural representations of drugs are post-processed to capture features of drug pairs associated with DDIs events [45]. Moreover, pharmacological properties such as targets [8], [46], enzymes, transporters, genes and proteins [6], [47], interaction pathways like enzymes and transporters [48], [49], [50], [51], [52], [53], [54], [55], [56], [57], [58], [59], [60], [61] can also be manipulated to represent drugs features through a set of descriptors. Network interaction mining [62], [63], [64] and molecular graph representations have also been used to describe substructures of drugs that come in distinctive shapes and sizes or the structural relations between entities [65], [66], [67], [68]. Additionally, to overcome the lack of data overlap between chemical content and biological characteristics, the combined structure-based input that includes both chemical and biological data by hybridizing cheminformatics and bioinformatics techniques to link all chemical information and biological effects have also been applied to serve as a meaningful method for DDIs discovery in many studies [69], [70], [71].

Many techniques have also been applied to cover multi pharmacological facets of DDI by admitting heterogeneous characterizations from various data sources that represent different drug characteristics and physiological effects [72], [73], [74]. The knowledge graphs (KGs)–based features integrated from multiple sources such as DrugBank, PharmGKB, and KEGG drugs [75] were used to overcome the limited information issue in single-source methods. Along with this, some efforts have been made to address the problem of increased noise in the integrated similarity. The similarity selection heuristic process ranks matrices based on the entropy calculated in each matrix and calculates their pair-wise distance for the final selection based on redundancy minimization [76], [77].

The classification feature constructing step usually requires the similarity analysis of paired drugs. In most studies, the chemical structural similarity was measured using the structures of the compound of drugs on DrugBank represented by their SMILES [6]. Structural representation of the drugs can be constructed using different molecular fingerprints generation techniques. The principle of this technique is to represent a molecule as a bit vector that codes the attendance or non-attendance of specifically assigned bit position structural features. Similarity measurements between molecular fingerprints are calculated using different methods; one commonly applied technique uses the Tanimoto coefficient [8], [48], [78]. Besides, many studies combine various drug-drug similarity measures representing relations between chemical, molecular physiological, or target pathways of drugs for the DDIs prediction task to gain more helpful information about DDIs [79], [80]. On the other hand, the network-based features processing method exploits the topological properties of the DDI network. Node2vec for Feature Network (FN) construction was used in [81] to present drug features as low-dimensional feature vectors.

## 4 Conventional ML-based prediction models of DDIs

Given the advanced computer science development and growing network pharmacology approaches, the development of a traditional ML-based model using multi-dimensional drug properties has been widely applied as a promising strategy to predict unknown DDIs [82], [83].

### 4.1 Single ML algorithm-based predictive model

Support vector machine (SVM) was a common algorithm used to predict DDIs due to its high performance with a broad range AUC value of 0.565 – 0.985 [19], [54], [6], [84], [85], [86], [87]. Indeed, the number of recruiting features has a certain role in the predictive model, e.g., a study applied the features reducing method and achieved an increase of 0.02 in the F-measure score (0.5786 vs 0.5965) of the predictive model [86]. Kernel machines are a class of algorithms for pattern analysis whose best-known member is the SVM. Kernel classifiers were used for classifying the drug pairs, including all-paths graph (APG), k-band shortest path spectrum (kBSPS), and the shallow linguistic (SL) kernel [17], [31], [88], [89]. Noteworthy, Thomas et al. [17] showed that SL and APG outperformed other methods, such as case-based reasoning and ensemble learning based on F1-score (0.606 vs. 0.416 and 0.583, respectively). Also, Zhang et al. [90] used the label propagation algorithms to work with the scenario where only a small portion of nodes in the undirected weighted network being labeled. In the meantime, logistic regression (LR) algorithm has been less used to establish DDIs prediction model. Xie et al. [91] integrated active learning, random negative sampling, and uncertainty sampling in clinical safety DDI information retrieval (DDI-IR) analysis using SVM and LR. In addition, Drug-Entity-Topic (DET) model following Bayes-rules was an example in leveraging augmented text-mining features to improve prediction performance in terms of discrimination and calibration [73]. Due to the growing demand for adverse DDIs (ADDIs) signal detection, Bayesian network framework and domain knowledge were combined to identify direct associations between a combination of medicines and the target ADEs [92]. Furthermore, gradient boosting-based algorithm XGBoost was employed to achieve robust DDI prediction even for drugs whose interaction profiles were completely unseen during training [60]. XGBoost performed better or comparable to other algorithms, such as SVM, random forest, and the standard gradient boosting in terms of predictive performance and speed in DDIs prediction [49], [60].

### 4.2 Ensemble learning predictive model

Ensemble methods use multiple learning algorithms to obtain better predictive performance than separate models in DDIs prediction [17], [33], [48], [72], [93], [94]. Combined ML algorithms using LibLINEAR, which consists of linear SVM, Naïve Bayes, and Voting Perceptron classifiers, outperformed the original (unbalanced) train corpora model based on F-score (70.4% vs. 69.0%)[95]. Similarly, a heterogeneous network-assisted inference (HNAI) framework consisting of five different ML algorithms, including Naive Bayes (NB), decision tree (DT), k-nearest neighbors (k-NN), LR, and SVM, was proposed to detect the unknown DDIs with AUC of 0.67, higher than that of separated algorithms (NB:0.66, DT:0.565, k-NN:0.6, LR:0.655, and SVM:0.666) [6]. Other ensemble methods including genetic algorithm and LR in classifier ensemble rule for DDIs prediction could obtain AUC value up to 1 and accuracy>90%, regardless of approved and unproved drug pairs being selected [48]. One of the significant concerns for developing a high-accuracy DDIs prediction model is integrating heterogeneous drug features. Thus, Zhang et al. [62] proposed a multi-modal deep auto-encoders based drug representation learning method (DDI-MDAE) to predict DDIs from large-scale, noisy and sparse data. DDI-MDAE encompasses RF classifier in the positive-unlabeled learning setting. Another computational experiment established a sparse feature learning ensemble method with linear neighborhood regularization (SFLLN) to predict DDIs, even unknown DDIs. Although SFLLN presented high accuracy and outperformed benchmark methods, it costs a reasonable amount of running time [70].

## 5 Deep learning-based prediction model of DDIs

As many as the number of drugs have entered the market over the past decades, the deep and complex interactions between drugs can go far beyond the capacity of simple traditional ML algorithms [96]. Therefore, DL, with multiple processing layers-concepts, is applied in DDIs prediction due to its ability to deal with complex relations [97]. Inspired by the architecture of human brains [98], the superior performance of DL in classification tasks over conventional methods leverages its growing application in DDIs prediction. Unlike the traditional ML method, which depends on hand-crafted features engineering, DL performed the data representation and prediction in a joint task. In a complex, ill-defined, and highly nonlinear problem as DDIs prediction, DL emerges as a suitable approach for solving these stochastic issues. DL can be seen as representation learning, in which the machine, which involves multiple sequential layers, can develop its feature representations [99]. We devoted this section to describing all leading DL frameworks in the DDIs extraction and prediction tasks since DL entered the field.

### 5.1 Artificial neural network (ANN)

ANN is a data-driven algorithm that seeks hidden functional relations from the dataset. In ANN, many neurons are connected in complex interconnections to solve linear or nonlinear problems. Previous studies have successfully manipulated ANN models for DDIs prediction tasks [100], [101]. The two layers ANN model has been used in the study of Rohani *et al.* [77] to work on a feature set of different similarity matrices collected from five different data sources. Masumshah *et al.* [102] used a feed-forward neural network with fully connected layers and the ReLU activation function was used between layers of the model as a sigmoid activation function for the output layer. Additionally, Shtar et al. [101] applied the ANN and propagation method over DDI graph nodes represented by an adjacency matrix. They used an XGBoost classifier for the DDIs classification, which output a binary value representing whether there is an interaction between the drug pairs or not.

### 5.2 Convolutional neural network (CNN)

CNN, which was inspired by the pattern of the animal Visual Cortex [103], has been introduced as an effective approach to deal with data with a grid pattern. The main goal of CNN is to transform the input into an easy-to-process form without compromising the prediction power. This characteristic makes CNN a potential candidate for the DDIs extraction task [104], [105] that requires valuable feature learning aspects and massive datasets scalability. The central concept of CNNs utilizes hidden convolution and pooling layers to identify spatially localized features via a set of receptive fields in kernel form. Usually, a CNN architecture consists of convolution, pooling, and fully connected layers. According to the task, it is also essential to have a suitable activation function. For example, a sigmoid function is often used in binary classification, while the softmax function is often applied in multiclass classification [106]. Different forms of CNN have been proposed for DDI prediction as follows.

#### 5.2.1 Conventional CNN

Chen *et al.* [107] used the CNN in the feature fusion module of their model, which was designed using a bi-level strategy with cross-and-scalar-level units. The CNN was used to learn the local and global features in the cross-level unit. The element-wise product was used in the scalar-level unit to get the fine-grained interactive feature between two features. These features will be concatenated to predict DDIs in the classifier module. The method proposed by Wu *et al.* [108] adopted two CNNs and the maximum pooling operation to extract features in the two location features from the word features preprocessed by the attention mechanism with a recurrent neural network (RNN). These features were then before fed into a softmax function to get the normalized probability score for each class. The model of Quan *et al.* [109] takes a DDIs instance represented by the word embedding and feeds them into the convolutional layer to get the filtered features. Then, the max-pooling layer extracts the essential local features; this layer also helps reduce the complexity of the model by reducing the feature dimension. Finally, in this model, a softmax layer is used for classifying DDIs types.

#### 5.2.2 Dependency-based CNN

The process of feeding local information into convolution operation in traditional CNN is not practical considering the case of long-distance relationships between words in candidate DDIs instances. Attempts to enlarge the window can lead to the data sparsity problem. Therefore, the dependency-based convolutional model (Dep-CNN) has been applied to capture long-distance dependencies between words of a sentence and extract DDIs from candidate instances. Dep-CNN performs convolution operation on adjacent words in word sentences and dependency parsing trees of candidate DDIs instances. In the model proposed by Liu *et al.* [110], they first generate a dependency parsing tree where each node corresponds to a word in the instance and syntactic dependency between two words denoted by the directed edge. Their Dep-CNN model is a four-layer neural network, consisting of a look-up table layer, a convolutional layer, a max-pooling layer, and a softmax progressing layer to feed the feature vector to a fully connected neural network for classification.

#### 5.2.3 Deep CNN

Considering various properties in texts, the successful application of Deep CNN (DCNN) in identifying complex patterns of image and video in computer vision [111] suggested its application in DDIs extraction task. Sun *et al.* [112] proposed a DCNN model which utilized a small convolution architecture to operate directly at the word level of the raw biomedical text input to get the embedding-based convolutional features. Then, the softmax classifier will be used to operate these features and extract DDIs from biomedical literature.

### 5.3 Graph convolutional neural network (GCNN)

In many DDIs prediction approaches, the molecular structure of drugs has been extensively exploited to extract the characteristics of the drug that link to the DDIs events. In non-Euclidean domains, where complex relationships and interdependencies between molecular structure representation of drugs or interactions between drug targets betokened as graphs [113], the application of GCNN in DDIs prediction was introduced. The most fundamental part of a GCNN is a graph, a data structure consisting of two components: nodes and edges [101]. The nodes usually represent the drug and edges are associated with interactions between nodes [114]. The first graph convolutional network was proposed by Bruna *et al.* [115] for applying neural networks to graph-structured data. Also, a model called SC-DDIS was introduced by Liu *et al.* [74] can learn the final embedding of drugs via a graph spectral CNN. Besides, it deals with the multiple complex structured entities that consist of two graph types: local graph for structured entities and global graph to capture structured entities' interactions. Wang *et al.* [85] proposed a graph to GCNN model called GoGNN to extract features in both graphs in a hierarchical fashion to leverage the DDIs prediction performance.

### 5.4 Recurrent neural network

RNN is highly manipulated in NLP [116], [117] and it mainly deals with sequential data. What makes RNNs differ from CNNs is their memory mechanism that gets information for the prior inputs to influence the current input and output. The DDIs extraction task is considered a relation extraction task in NLP. Many have utilized the long short term memory (LSTM) network to extract DDIs from literature [118], [119], [120]. Even though Char-RNNs are more common for modeling morphologically richer languages [121] and were introduced for text classification [122]. Kavuluru *et al.* [123] has also considered the role of character-level embedding in DDIs extraction, and they used an LSTM on the character embedding to extract the word vectors.

Luo *et al.* [57] presented a model that used an LSTM model for DDIs prediction in diabetes using the embedded drug-induced transcriptome data. The LSTM is a typical RNN architecture introduced by Hochreiter and Schmidhuber [124] to deal with the problem of long-term dependencies. In LSTM, cells in the hidden layers contain an input gate, an output gate, and a forget gate to control the flow of information required for the Prediction. Also, the gated recurrent units (GRU) was introduced to address the short-term memory problem of the RNNs model [125]. However, unlike the LTSM, GRUs use hidden states and two gates: reset and update gate to control the information to retain for the prediction.

For the DDIs extraction task, a hierarchical RNN was introduced by Zhang *et al.* [33]. This model framework considers the shortest dependency path (SDP) between two entities and uses the RNN to learn the feature representation of sentence sequence and SDP for extracting DDIs. Zhou et al. [126] introduced an attention-based BiLSTM model to encode biomedical text sentences.

Besides, considering the difference between DDIs instance and typical sentence, Jiang *et al.* [127] used a skeleton structure to represent the DDIs instances and the LSTM model to work with the structure (skeleton-LSTM). In their framework, a sentence is first tokenized into token units followed by a corresponding skeleton unit, distance to the first drug, and distance to the second drug. These units are input to the embedding layer of the skeleton-LSTM.

However, traditional Encoder-Decoder architecture using RNN or LSTM remained several drawbacks as it can cause the information loss problem, especially in the case of long sentences. Attention mechanism has been applied to deal with the problem mentioned above [128]. The model proposed by Yi *et al.* [129] used a bidirectional RNN layer to generate a sentence matrix as the word's semantic representation. Then, the attention layer is applied to create the final representation by combining several relevant sentences of the same drug pairs. The softmax classifier was used to classify specific DDIs. Zheng *et al.* [130] also introduced a model to classify DDIs from texts using a combined attention mechanism and an RNN with LSTM units.

## 6 Interpretability methods in XAI and XAI in DDIs prediction

The surge in the predictive performance of AI tools is achieved by increasing model complexity. This turns these models into black-box systems and causes uncertainty regarding their operation mechanism. This ambiguity hinders the wide adaptation of AI models in critical domains like healthcare. As a result, eXplainable Artificial Intelligence (XAI) focuses on understanding behind the prediction of AI models to accommodate the demand for transparency in AI tools. Interpretability methods of AI models can be classified based on the type of algorithms, the interpretation scale, and the data type [131]. Additionally, based on the purposes of interpretability, approaches can be categorized as white-box models creation, black-box models explanation, enhancement of model fairness and predictive sensitivity testing [132].

In terms of methods to explain DL models, the gradient-based attribution method [133] attempts to explain the prediction by attributing them to the network's input features. This method is often applied when predictions are made from a DNN system and therefore, can be potential approach for some black-box DNN models in DDIs prediction like [110], [112]. Moreover, the DeepLIFT is a popular algorithm applied on top of DNN models that showed considerable advantages compared to gradient-based methods [134]. On the other hand, Guided BackPropagation method can be applied to network structures [135]. Under this, a convolutional layer with improved stride can replace max-pooling in CNN to deal with accuracy loss. This approach suggests a potential application in some CNN-based DDIs prediction such as [111]. On top of this, the [136] was proposed in NLP-based neural networks. This method used rationales (small pieces of input text) and tried to produce the same prediction as the full-text input type. Under this method, the architecture consists of two components, generator and encoder, to look for text subsets highly related to the prediction result. Since the DDIs extraction task is conducted via NLP-based models [109], [114], the above methods should be considered for application to promote the clarity of these models.

Apart from this, methods to create white-box models such as linear, decision tree, rule-based models, or sophisticated yet transparent models have also been proposed in XAI. However, due to the limited predictive power, especially in the NLP-based domain as in the DDIs extraction task, these approaches are given less interest. Additionally, various methods have been proposed to tackle fairness in AI. Nevertheless, a minimal number of these scientific pieces of literature considered fairness in non-tabular data such as text-based information for DDIs extraction. While many DDIs studies applied the word embedding method [62], [109], it was revealed that vectorized representing of text data could carry strong bias [137]. Therefore, methods to assure fairness should be taken into more consideration in DDIs studies. Furthermore, some methods aim to analyze the sensitivity of AI models to ensure the reliability of those tools. In the Adversarial Example-based Sensitivity Analysis, Zugner *et al.* [138] used this approach to study the graph-structured data. This method considers modifying node connections or node features to attack node classification models. Since graph-based methods are widely applied in DDIs studies [67], [68], approaches as in the above research suggest potential application in DDIs prediction model. Also, using perturbations to the word embeddings [139] in RNN should also be considered. Significantly, the input reduction method in the study of Feng *et al.* [140] to reveal oversensitivity in NLP models can be a possible approach in DDIs extracting studies. Literature regarding the explosion of the weakness of DL models in NLP-tasks is complete; however, applications in DDIs- NLP models are still limited.

In the DDIs study of Schwarz *et al.* [61], an attempt has been made to offer their model interpretability using the Attention scores computed at all layers of modeling. Using these scores, the contribution of the similarity matrices to the drug representation vectors is determined and the drug characteristics that lead to better encoding are selected. This approach leverages information that passes through all layers of the network.

## 7 Challenges and opportunities

Though traditional ML performed effectively in extracting DDIs, even from the unstructured package insert (aka drug product label) [87], conventional ML-based methods still have several drawbacks. ML-based models are learned from positive and negative data, making it difficult in real-world domains due to the lack of true negative DDIs or a “gold standard” non-DDI. Therefore, it is necessary to identify positive data from many unlabeled data containing positive and negative samples and avoid biased sampling by random negative sampling and validation set updating. Additionally, it is unknown whether there is DDI between two drugs in a negative class dataset because some new DDIs drug pairs may not be reported yet. Another issue is different types of DDI data, such as clinical drug safety and pharmacokinetic data with different targeted samples and proportions in DDI-relevant databases or articles. Also, it is more time-consuming to accomplish the annotated corpora and determine optimal parameters in traditional ML-based methods. Hence, DNN models, including CNN and sequential neural networks such as RNN, have been referred to as an optimal resolution for feature selection and DDIs extraction without complicated feature engineering [120]. However, we assumed that several paths should be investigated in future work. First, drug-related textual data sources, such as patent information, are essential. Second, it is unknown how to use drug domain knowledge or semi-structured drugs, such as paragraph that describes the pharmacodynamics or mechanism of action, protein binding, or experimental properties of a drug in building up predictive models.

In addition, DL with superior performance and capability to automatically generate hierarchical input for the classification tasks has gained huge research attention in DDIs prediction domain. Still, these DL methods are neither easily explainable nor commonly trusted by medical staff because of their explainability deficiency. In the DDIs prediction field, only a few studies have considered the explainable aspect of their models, which leaves plenty of room to improve, innovate, and ensure predictive performance and model interpretability in ML-based DDIs prediction models. We, therefore, think that either approaches to explain black-box models, methods to create high-accuracy white-box models, strategies to ensure models fairness, or strict sensitivity analyses of models in DDIs prediction should be given more consideration in the coming years to produce trust and fairness in these models' performance and bring them closer to clinical application. Since XAI aims to explain the machine learning models, its application does not lead to less accuracy in current models. Also, further studies can show the potential of XAI in sacrificing accuracy in the field of DDIs extraction task (NLP) if text based approach is usually used for replenishment of databases and one can refine the found dependencies in the initial sources. Addressing it may open a new road in the application of XAI in DDI prediction in the future, especially for DDI extraction task using NLP.

## 8 Conclusion

The management of DDIs, which can cause ADEs and affect patients' health, plays a crucial role in pharmacovigilance and medical practice. The main contribution of this study is the establishment of detailed taxonomy of existing models for predicting DDIs. Given remarkable breakthroughs in DDIs prediction over the past years, weakness in terms of model interpretability exposed considerable limits. We, therefore, believe that XAI in DDIs prediction still holds many potential aspects to unlock in future studies.

## CRediT authorship contribution statement

**Thanh Hoa Vo:** Conceptualization, Methodology, Formal analysis, Data curation, Writing – original draft, Writing – review & editing, Visualization. **Ngan Thi Kim Nguyen:** Methodology, Formal analysis, Validation, Writing – original draft, Writing – review & editing, Visualization. **Quang Hien Kha:** Validation, Data curation. **Nguyen Quoc Khanh Le:** Conceptualization, Methodology, Formal analysis, Investigation, Data curation, Writing – original draft, Writing – review & editing, Visualization, Supervision, Funding acquisition.

## Declaration of Competing Interest

The authors declare that they have no known competing financial interests or personal relationships that could have appeared to influence the work reported in this paper.
